# *Malat1* Suppresses Immunity to Infection through Promoting Expression of Maf and IL-10 in Th Cells

**DOI:** 10.4049/jimmunol.1900940

**Published:** 2020-04-22

**Authors:** James P. Hewitson, Katie A. West, Kylie R. James, Gulab Fatima Rani, Nidhi Dey, Audrey Romano, Najmeeyah Brown, Sarah A. Teichmann, Paul M. Kaye, Dimitris Lagos

**Affiliations:** * York Biomedical Research Institute, University of York, York, YO10 5DD Yorkshire, United Kingdom;; † Department of Biology, University of York, York, YO10 5DD Yorkshire, United Kingdom;; ‡ Hull York Medical School, University of York, York, YO10 5DD Yorkshire, United Kingdom;; § Wellcome Sanger Institute, CB10 1SA Hinxton, United Kingdom;; ¶ Theory of Condensed Matter, Cavendish Laboratory, Department of Physics, University of Cambridge, CB3 0HE Cambridge, United Kingdom; and; ‖ European Molecular Biology Laboratory, European Bioinformatics Institute, CB10 1SA Hinxton, United Kingdom

## Abstract

Despite extensive mapping of long noncoding RNAs in immune cells, their function in vivo remains poorly understood. In this study, we identify over 100 long noncoding RNAs that are differentially expressed within 24 h of Th1 cell activation. Among those, we show that suppression of *Malat1* is a hallmark of CD4^+^ T cell activation, but its complete deletion results in more potent immune responses to infection. This is because *Malat1^−/−^* Th1 and Th2 cells express lower levels of the immunosuppressive cytokine IL-10. In vivo, the reduced CD4^+^ T cell IL-10 expression in *Malat1^−/−^*mice underpins enhanced immunity and pathogen clearance in experimental visceral leishmaniasis (*Leishmania donovani*) but more severe disease in a model of malaria (*Plasmodium chabaudi chabaudi* AS). Mechanistically, *Malat1* regulates IL-10 through enhancing expression of Maf, a key transcriptional regulator of *IL-10*. Maf expression correlates with *Malat1* in single Ag-specific Th cells from *P. chabaudi chabaudi* AS–infected mice and is downregulated in *Malat1^−/−^* Th1 and Th2 cells. The *Malat1* RNA is responsible for these effects, as antisense oligonucleotide-mediated inhibition of *Malat1* also suppresses Maf and IL-10 levels. Our results reveal that through promoting expression of the Maf/IL-10 axis in effector Th cells, *Malat1* is a nonredundant regulator of mammalian immunity.

## Key Points

- *Malat1* is suppressed during Th1 and Th2 differentiation.
- *Malat1* loss suppresses IL-10 and Maf expression in effector Th cells.
- *Malat1^−/−^* mice mount enhanced immune responses in leishmaniasis and malaria models.

## Introduction

Long noncoding RNAs (lncRNAs) are >200-nt transcripts that lack protein-coding potential but have regulatory functions (1, 2). Mammalian genomes contain thousands of lncRNAs and demonstrate the highest frequency in lncRNA transcripts compared with any other species (1). These are mostly medium to lowly expressed transcripts, displaying poor conservation across mammals. Their modes of action vary, but they often act as scaffolds, recruiting or sequestering chromatin-modifiers or RNA-binding proteins (RBPs) to specific genomic sites (2). Despite remarkable progress in mapping lncRNAs to mammalian genomes and exploring lncRNA function at the molecular level in cellular systems, there is a profound lack of understanding of the function of lncRNAs (requirement, sufficiency, or redundancy) at the whole-organism level. For example, although CD4^+^ Th cells are central to pathogen-specific adaptive immunity (3), and there are hundreds of lncRNAs identified as differentially regulated during CD4^+^ T cell activation in humans and mice (4–6), fewer than a handful of lncRNAs have been shown to affect Th cell function. These include *NeST* (7), which has been shown to control its neighboring *Ifng* locus, and *lincR-Ccr2-5′ AS* (5) and *linc-Maf-4* (6), which affect CD4^+^ T cell gene expression through long-range interactions. Therefore, the functional relevance of lncRNAs in vivo is a largely unexplored and emerging challenge in both the fields of immunology and RNA biology.

Metastasis-associated lung adenocarcinoma transcript 1 (*Malat1*) is a 7.5-kb-long long intergenic noncoding RNA (lincRNA) transcript, which is associated with cancer progression and metastasis (8). It is localized in nuclear speckles (9), which are nuclear foci enriched in factors involved in pre-mRNA splicing and transcription (10). In contrast to the vast majority of lncRNAs, *Malat1* is highly conserved across mammals and highly and ubiquitously expressed (5,000–10,000 copies per cell). It has been somewhat surprising that characterization of three independent *Malat1* knockout (*Malat1*^−/−^) mouse models did not reveal any homeostatic phenotypes (abnormal development, viability, fertility, or lifespan) or any patent defects in nuclear architecture (e.g., speckle formation) (11–13). In the context of CD4^+^ T cell function, two recent reports presented contradicting results regarding *Malat1* function; Yao and colleagues (14) found that *Malat1* does not affect number of CD4^+^ T cells and T follicular helper cells or CD8^+^ T cells responses to lymphocytic choriomeningitis virus (LCMV) in vivo and concluded that *Malat1* is dispensable for CD4^+^ T cell function and development, whereas Masoumi and colleagues (15) reported that *Malat1* is downregulated in tissues from patients with multiple sclerosis and mice with experimental autoimmune encephalomyelitis and that small interfering RNA–mediated knockdown of *Malat1* promoted Th1/Th17 polarization and inhibited T regulatory cell differentiation in vitro. The above demonstrate that the physiological function of *Malat1* in vivo and potential role in adaptive immunity remain poorly understood.

In this study, through defining the lncRNA signature of early Th cell activation, we show that *Malat1* is one of the most highly abundant transcripts in naive CD4^+^ T cells and it is downregulated within the first 24 h of naive CD4^+^ T cell activation. Suppression of *Malat1* expression is sustained and observed in in vitro–differentiated Th1 and Th2 cells. Single-cell RNA sequencing (RNA-seq) analyses of in vivo–derived Ag-specific Th1 cells demonstrate that *Malat1* expression inversely correlates with expression of transcriptional units involved in RNA processing and translation, protein degradation, metabolism, and cellular structure, all hallmarks of Th activation. Similar correlations are seen in Th2 cells. Conversely, *Malat1* expression positively correlates with expression of *Maf* (also known as c-Maf). Functionally, when compared with wild-type (WT) C57BL6 controls, in vitro–generated *Malat1*^−/−^ Th1 and Th2 cells express lower levels of Maf and its transcriptional target IL-10. Suppression of IL-10 expression in *Malat1*^−/−^ CD4^+^ T cells is also observed in mice infected with the protozoan parasite *Leishmania donovani* or with *Plasmodium chabaudi chabaudi* AS (*Pc*AS). *Malat1*^−/−^ mice demonstrate enhanced macrophage activation and parasite clearance in the visceral leishmaniasis model, but more pronounced disease in experimental malaria, similarly to phenotypes observed in IL-10–deficient mice. Overall, our results demonstrate that *Malat1* suppression is a hallmark of CD4^+^ T cell activation and controls IL-10 expression in Th cells. We propose that suppression of *Malat1* in activated CD4^+^ T cells is a critical determinant of optimal immunity to chronic infection.

## Materials and Methods

### Ethics

Animal care and experimental procedures were regulated under the Animals (Scientific Procedures) Act 1986 (revised under European Directive 2010/63/EU) and were performed under U.K. Home Office License (project license number PPL 60/4377 with approval from the University of York Animal Welfare and Ethical Review Body). Animal experiments conformed to Animal Research: Reporting of In Vivo Experiments guidelines.

### Mouse infections

Female C57BL/6 CD45.2 mice were obtained from Charles River Laboratories. *Malat1*^−/−^ mice (complete knockouts) were obtained from the Riken Institute (12). All mice were bred in-house, maintained under specific pathogen-free conditions, and used at 6–12 wk of age. The Ethiopian strain of *L. donovani* (LV9) was maintained by passage in RAG-2^−/−^ mice. Mice were infected i.v. with 30 × 10^6^ amastigotes via the tail vein. Parasite burden was expressed parasite count per 100 host cell nuclei or as Leishman–Donovan units (the number of parasites per 1000 host cell nuclei × organ weight in milligrams).

Female C57BL/6 or *Malat1*^−/−^ mice (6–12 wk old) were infected with *Pc*AS through i.v. injection of 1 × 10^5^ parasitized erythrocytes under reverse light cycles. Parasitemia was monitored from day 5 onwards by thin blood smears stained with Giemsa stain (infected red cells per 1000 red cells × 100). Mice were bled within the first 2 h of the dark cycle. Weights and signs of disease were monitored daily.

### FACS analysis and cell sorting

For FACS analysis, spleens were first digested with 0.4 U/ml Liberase TL (Roche) and 80 U/ml DNase I type IV in HBSS (both Sigma-Aldrich) for 15 min at 37°C. Enzyme activity was inhibited with 10 mM EDTA (pH 7.5) and single-cell suspensions were created with 70-μm nylon filters (BD Biosciences) in complete RPMI 1640 (Thermo Fisher Scientific) supplemented with 10% heat-inactivated FCS (HyClone), 100 U/ml penicillin, 100 μg/ml streptomycin, and 2 mM l-glutamine (all Thermo Fisher Scientific). RBCs were lysed with RBC lysing buffer (Sigma-Aldrich). For live/dead discrimination, cells were washed twice in PBS, then stained with Zombie Aqua (BioLegend) before resuspension in FACS buffer (PBS containing 0.5% BSA and 0.05% azide). Fc receptors were blocked with 100 μg/ml rat IgG (Sigma-Aldrich) for 10 min at 4°C before surface staining for 30 min at 4°C. Combinations of the following anti-mouse Abs were used: CD45.1 allophycocyanin (clone A20); CD45.2 BV786 (104); CD3 FITC (145-2C11); B220 FITC (RA3-6B2); TCRβ PE-Cy7 (H57-597); MHC class II (MHCII) Alexa Fluor 700 (M5/114.15.2); Ly-6G PE-Cy7 (1A8); CD11b Pacific Blue and allophycocyanin (M1/70); CD11c PerCP/Cy5.5 (N418); F4/80 FITC and Alexa Fluor 647 (BM8); CD44 FITC (IM7); CD62L PE (MEL-14); CD8α allophycocyanin (53-6.7); CD4 PE and PerCP/Cy5.5 (RM4-5); IFN-γ FITC (XMG1.2); IL-10 PE (JES5-16E3); and IL-17A PE/Cy7 (TC11-18H10.1). All Abs were from BioLegend. To measure intracellular cytokines in T cells following ex vivo stimulation, cells were first stimulated in complete RPMI 1640 for 4 h at 37°C with 500 ng/ml PMA, 1 μg/ml ionomycin, and 10 μg/ml brefeldin A (all Sigma-Aldrich). For all intracellular cytokine staining, surface stained cells were fixed and permeabilized (20 min at 4°C) using Fixation/Permeabilization Solution before washes in Perm/Wash buffer (both BD Biosciences). Cells were then stained with intracellular Abs as above except in Perm/Wash buffer. Appropriate isotype controls were included. For FACS analysis, events were acquired on an LSRFortessa (BD Biosciences) before analysis with FlowJo (FlowJo). For cell sorting of splenic lymphocytes from naive and *L. donovani*–infected spleens, CD4^+^ T cells were sorted as B220^−^ CD3^+^ CD4^+^ CD8a^−^. For purification of naive and activated CD4^+^ T cells from uninfected mice, single-cell suspensions were prepared from pooled spleens and peripheral lymph nodes (axillary, brachial, and inguinal). CD4^+^ cells were enriched using CD4 microbeads and LS columns (Miltenyi Biotec) before cell sorting of naive CD4^+^ T cells (CD4^+^ CD62L^+^ CD44^−^ CD11b^−^ CD8a^−^ MHCII^−^). Cell sorting was performed with a MoFlo Astrios (Beckman Coulter), and sorted cells were typically >98% positive.

### In vitro activation of CD4^+^ T cells

Purified CD4^+^ T cells were stimulated with 10 μg/ml plate-bound anti-CD3ε (clone 145-2C11) and 2 μg/ml soluble anti-CD28 (37.51) in RPMI 1640 as before in flat-bottom 96-well plates (Th0 conditions). For Th1 polarization, cells were also treated with 15 ng/ml mouse rIL-12 and 5 μg/ml anti–IL-4 (11B11) or, for Th2 polarization, 30 ng/ml mouse rIL-4 and 5 μg/ml anti–IFN-γ (XMG1.2). To induce suboptimal Th1 differentiation (weakly polarizing conditions), 1% of the original stimulation concentrations of recombinant cytokine rIL-12 and anti–IL-4 were included in the cell culture medium. To induce suboptimal Th2 differentiation, 2% of the original concentrations of rIL-4 or anti–IFN-γ were included in the cell culture medium. Anti-CD3/anti-CD28–dependent activation (4 d) was followed by rest in 10 U/ml human rIL-2 (2 d). To induce Th17 differentiation, naive CD4^+^ T cells were stimulated with 10 ug/ml plate-bound anti-CD3 (145-2c11) and 4 μg/ml soluble anti-CD28 (37.51), and 1 ng/ml of rTGF-β, 37.5 ng/ml rIL-6, 5 μg/ml anti–IFN-γ (XMG1.2), and 5 μg/ml anti–IL-4 (11B11). After 3 d of stimulation, cells were transferred to a new 96-well plate in the presence of half the concentration of recombinant cytokines and inhibiting Abs. Cells were harvested and analyzed by flow cytometry at day 5. All Abs were from BioLegend and were low on endotoxin and azide free. Recombinant cytokines were from PeproTech. Control or *Malat1*-targeting antisense oligonucleotide gapmers were from QIAGEN (Hilden, Germany; LG00000002-DDA and LG00000008-DDA, respectively) and were added to naive CD4^+^ T cells on day 0 at a final concentration of 100 nM.

### Quantitative RT-PCR

RNA was extracted from tissue samples or purified cell populations using QIAzol and miRNeasy RNA extraction kits (QIAGEN) according to manufacturer’s instructions. Tissue samples were first dissociated in QIAzol using a TissueLyser LT with stainless steel beads (all QIAGEN). For mRNA transcripts, reverse transcriptions were carried out with Superscript III (Thermo Fisher Scientific) and random hexamer primers (Promega) and measured with Fast SYBR Green Master Mix (Thermo Fisher Scientific). PCR were performed using a StepOnePlus Real Time PCR System (Thermo Fisher Scientific), and relative transcript levels were determined using the ∆∆Ct method. The following primer sequences were used: *Malat1* forward, 5′-TGCAGTGTGCCAATGTTTCG-3′; *Malat1* reverse, 5′-GGCCAGCTGCAAACATTCAA-3′; *Neat1* forward, 5′-CCTAGGTTCCGTGCTTCCTC-3′; *Neat1* reverse, 5′-CATCCTCCACAGGCTTAC-3′; U6 forward, 5′-CGCTTCGGCAGCACATATAG-3′; and U6 reverse, 5′-TTCACGAATTTGGCTGCTAT-3′.

For all other genes commercially available, QuantiTect (QIAGEN) primer sets were used.

### RNA-seq analysis

For single-cell RNA-seq analyses, Smart-seq2 single-cell RNA-seq FASTQ files were obtained from ArrayExpress (www.ebi.ac.uk/arrayexpress/), accession numbers E-MTAB-4388 (Th1) and E-MTAB-2512 (Th2). Sequencing reads were mapped to the mouse mm10 Ensembl 84 reference genome with External RNA Controls Consortium RNA spike-in sequences using star 2.5.1b and quantified with HTSeq 0.9.1. Normalization and filtering of data were performed using Seurat (version 3.0.0). Cells with expression of fewer than 200 genes and/or a mitochondrial read proportion above 5% were excluded from analysis. Genes were filtered for minimum expression in three cells. Gene correlations were determined from log-normalized expression data using cor.test function (stats package) with Spearman rho statistic to estimate a rank-based measure of association.

Bulk RNA-seq analyses were performed as previously described (16). Briefly, we compared four naive CD4^+^ T cells against four activated (24 h) samples. We obtained 10 million reads per sample on average (range: 4–20 million). Sequence reads were trimmed to remove adaptor sequences with Cutadapt and mapped to mouse genome GRCm38 with HISAT2, including rna-strandness FR option. Transcriptome assembly and quantification was performed using the Tuxedo pipeline (version 2.2.1). Cufflinks was used to assemble transcriptomes for each sample using the Gene transfer format annotation file for the GRCm38 mouse genome. This was followed by running Cuffmerge to merge individual sample transcriptomes into full transcriptomes. Quantification and normalization were carried out for each experiment using Cuffquant and Cuffnorm. Differential expression on gene fragments per kilobase of transcript per million mapped reads (FPKM) values was performed by conducting paired and independent *t* tests with Benjamini–Hochberg false discovery rate correction. Data are available at Gene Expression Omnibus (https://www.ncbi.nlm.nih.gov/geo/) accession number GSE125268 (wild-type samples only). Gene ontology and Search Tool for the Retrieval of Interacting Genes/Proteins (STRING) analysis (http://string-db.org/) were performed where indicated. STRING settings were highest-confidence interactions only excluding text mining. Transcription factors and cofactors were extracted by comparing gene lists to the TFcheckpoint database (http://www.tfcheckpoint.org/).

### Western blotting

Cells were washed twice in PBS and protein extracts prepared in radioimmunoprecipitation assay buffer (150 mM NaCl, 10 mM Tris (pH 7.2), 5 mM EDTA, 0.1% SDS, 0.1% Triton X-100, 1% sodium deoxycholate, 1 mM PMSF, 1% protease inhibitor mixture P8340, and 1% phosphate inhibitor mixtures 2 and 3; all Sigma-Aldrich). Equal total amounts of protein were resolved on SDS-PAGE gels and transferred to polyvinylidene difluoride membranes (MilliporeSigma) using a Bio-Rad SD Semi-Dry Transfer Cell, blocked for 2 h at room temperature in 2% BSA (Thermo Fisher Scientific) or 5% milk powder (Sigma-Aldrich) in TBST (150 mM NaCl, 7.7 mM Tris HCl [pH 8], and 0.1% Tween 20; all Sigma-Aldrich) before overnight probing with primary Abs at 4°C. Abs were as follows: Maf (55013-1-AP) and Stat4 (51070-2-AP) from Proteintech, Histone 3 from Cell Signaling Technology. Following extensive washing in TBST, blots were incubated with secondary Abs (goat anti-rabbit or mouse HRP; DAKO) for 1 h at room temperature, washed as before, and developed with ECL Western Blotting Detection Reagent and Hyperfilm ECL (both GE Healthcare).

### Statistical analysis

Statistical analyses were carried out as indicated with Prism 5 (GraphPad Software). Two-way comparisons used paired or unpaired *t* tests as indicated, and multiple comparisons used one-way ANOVA, followed by Bonferroni correction for multiple testing. Any *p* values <0.05 were considered significant. All *p* values are shown when significant or borderline.

## Results

### *Malat1* is suppressed at the early stages of CD4^+^ T cell activation

**FIGURE 1.**
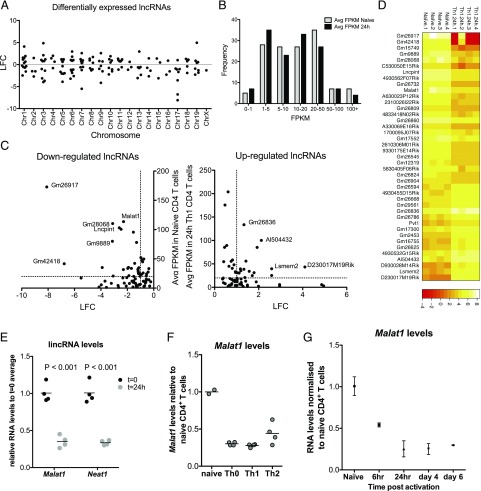
Identification of differentially regulated lncRNAs upon activation of naive CD4^+^ T cells reveals *Malat1* suppression as a hallmark of Th cell activation. (**A**) LFC of statistically significantly differentially regulated lncRNAs (false discovery rate < 0.05) per chromosome following 24 h activation of naive CD4^+^ T cells under Th1 conditions. Expression determined by bulk RNA-seq (*n* = 4). (**B**) Distribution of lncRNA expression in naive and activated (24 h, Th1 conditions) CD4^+^ T cells. (**C**) Expression versus LFC for all the differentially regulated lncRNAs. Dotted lines indicate LFC = 1 and FPKM = 20. (**D**) Expression heatmap of most highly expressed and differentially expressed lncRNAs. For downregulated, lncRNAs with LFC > 1 and naive CD4^+^ FPKM > 20 are shown. For upregulated, lncRNAs with LFC > 1 and Th1-activated CD4^+^ FPKM > 20 are shown. (**E**) Expression of *Malat1* and *Neat1* in naive and Th1-activated CD4^+^ T cells (24 h postactivation) determined by qRTPCR. Expression is normalized to U6 RNA and average expression in naive CD4^+^ T cells. (**F**) *Malat1* expression determined in in vitro–differentiated Th0, Th1, and Th2 cells (day 6). Expression is normalized to U6 RNA and average expression in naive CD4^+^ T cells. (**G**) *Malat1* expression during in vitro Th1 differentiation, normalized to naive CD4^+^ T cells.

To gain insight into the role of the noncoding transcriptome in CD4^+^ T cell function, we began our studies by using bulk RNA-seq to determine early (24 h) changes in expression of lncRNAs under in vitro Th1-polarizing conditions. We identified 120 differentially expressed lncRNAs, the majority of which were intergenic, distributed across the genome (Fig. 1A). As expression of lncRNAs varied (Fig. 1B), we set an additional criterion in our analysis and identified 23 lncRNAs that were expressed in CD4^+^ T cells at FPKM > 20 and were differentially regulated (log_2_ fold change [LFC] > 1 or LFC < −1) between naive and 24-h-activated CD4^+^ T cells (Fig. 1C, 1D). We observed a significant correlation between changes in expression of the identified lncRNAs and their chromosomally adjacent genes (Supplemental Fig. 1A), which inferred the existence lncRNA-mediated in cis regulatory effects across the CD4^+^ T cell transcriptome, in agreement with previous studies (1).

We further investigated the list of highly expressed and differentially regulated lncRNAs and noticed that *Malat1* (Fig. 1C) was the most highly expressed dysregulated lncRNA that was also conserved between the mouse and human genome (*Gm26917* and *Gm26836* were expressed at higher levels than *Malat1* but have no obvious sequence or syntenic homologs in the human genome). Of note, *Malat1* was within the top 2% most highly expressed transcripts in naive CD4^+^ T cells (Supplemental Fig. 1B). *Malat1* suppression was confirmed by quantitative RT-PCR (qRTPCR) (Fig. 1E). *Neat1*, an lincRNA adjacent to *Malat1* in both mouse and human genome, was also found to be significantly downregulated within 24 h of CD4^+^ T cell activation by qRTPCR (Fig. 1E). The downregulation of *Neat1* upon CD4^+^ T cell activation did not reach statistical significance in our RNA-seq analysis, likely because of higher variation and lower absolute expression of *Neat1* compared with *Malat1* in CD4^+^ T cells (Supplemental Fig. 1C). Suppression of *Malat1* and *Neat1* expression were also observed in end point (day 6) differentiated Th0-, Th1-, and Th2-polarizing conditions in vitro (Fig. 1F). Both *Malat1* and *Neat1* were downregulated within hours of naive CD4^+^ T cell activation (Fig. 1G, Supplemental Fig. 1D). The above results revealed that the rapid suppression of *Malat1* and *Neat1* expression is a key feature of the early lncRNA signature of CD4^+^ T cell activation.

### *Malat1* suppression is a transcriptomic hallmark of CD4^+^ T cell activation

**FIGURE 2.**
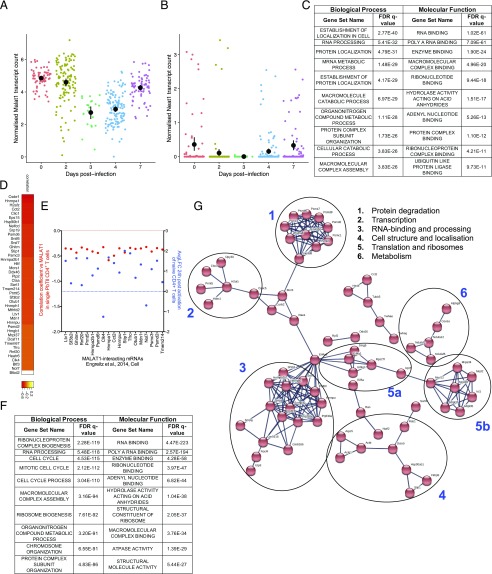
Transcriptional units and transcription factors correlating with *Malat1* expression at single-cell level in Th cells in vivo. (**A**) Normalized transcript counts of *Malat1* in single *Plasmodium*-specific CD4^+^ T (PbTII) cells isolated at the indicated timepoints following infection with *Pc*AS. Mean and 95% confidence intervals are shown. (**B**) Normalized transcript counts of *Neat1* in single PbTII cells isolated at the indicated timepoints following infection with *Pc*AS. Mean and 95% confidence intervals are shown. (**C**) Most highly enriched gene ontology (GO) terms among significantly negatively correlated (*p* < 0.05) genes with *Malat1* in PbTII cells isolated from *Pc*AS-infected mice (7 d p.i.). (**D**) Heatmap of correlation coefficients of *Malat1* and indicated *Malat1*-interacting mRNAs (21, cell) in single PbTII cells from *Pc*AS-infected mice 7 d p.i. (**E**) Correlation coefficient of *Malat1* and indicated *Malat1*-interacting mRNAs (left *y*-axis, red) in single PbTII cells from *Pc*AS-infected mice (7 d p.i.) versus average LFC following 24 h Th1 in vitro activation of naive CD4^+^ T cells (right *y*-axis, blue). Values are shown for significantly differentially expressed genes (FDR < 0.05) following 24 h Th1 activation of naive CD4^+^ T cells. (**F**) Most highly enriched GO terms among significantly negatively correlated (*p* < 0.01) genes with *Malat1* in single in vitro–differentiated Th2 cells. (**G**) STRING network of significantly correlated genes with *Malat1* at single-cell levels in both in vivo PbTII Th1 and in vitro Th2 cells. Functional clusters are numbered and shown.

To gain insight into whether *Malat1* suppression was associated with CD4^+^ T cell function, we first analyzed a single-cell RNA-seq dataset we previously published exploring emergence of Th1 cells in vivo (17) using *Plasmodium*-specific TCR transgenic CD4^+^ T (PbTII) cells transferred into congenically labeled mice and recovered at days 2, 3, 4, and 7 postinfection (p.i.) with *Pc*AS. In agreement with the observed suppression of *Malat1* in our in vitro experiments (Fig. 1), we observed *Malat1* and *Neat1* downregulation upon CD4^+^ T cell activation in vivo, reaching a minimum at days 3 and 4, followed by a relative increase by day 7 (Fig. 2A, 2B). As observed in vitro, *Neat1* expression was lower than *Malat1* in Th1 cells in vivo (Fig. 2A, 2B).

Next, we searched for transcriptomic units that show significant correlation with *Malat1* expression at the single-cell level. We performed these analyses using the data from PbTII CD4^+^ T cells at day 7 p.i. because this is the time point that the strongest Th1 responses are observed (17). Analyses were performed in gated Th1 cells based on IFN-γ and CXCR6 expression rather than all CD4 PbTII cells at this time point. We searched for genes, the expression of which was positively or negatively correlated with that of *Malat1*. Strikingly, we found that from 687 genes that demonstrated significant correlation with *Malat1* in Th1 cells, 609 (88.6%) correlated negatively (i.e., cells with low *Malat1* levels demonstrate high levels of these genes). We noted that this was not purely due to the fact that *Malat1* is downregulated in Th1 cells, as performing the same analysis for *Lncpint*, an lincRNA with similar basal expression in CD4^+^ T cells that is also downregulated upon activation (Fig. 1C) did not show a similar bias toward negative correlation (Supplemental Fig. 2A). *Neat1* was within the positively correlated genes (Supplemental Fig. 2B), consistent with reports showing that the two lincRNAs are coregulated (18). Gene ontology analysis of the negatively correlated genes revealed significant enrichment in genes involved in RNA binding, ribosomal function, metabolism, and cellular structure/localization (Fig. 2C). We noted that these were processes associated with cellular activation and specifically naive CD4^+^ T cell differentiation to effector Th cells (19, 20). Analysis of a published set of *Malat1*-interacting mRNAs in embryonic stem cells (21) indicated that *Malat1* has the potential to interact with the mRNA of 43 of the coregulated genes (Fig. 2D). Notably, 19 of these genes were also statistically significantly differentially expressed within 24 h of Th1 activation, with a dominant trend toward upregulation (Fig. 2E).

To assess whether the above *Malat1*-correlated gene signatures were specific to Th1 cells, we analyzed a single-cell RNA-seq dataset we previously published exploring gene expression in in vitro–polarized Th2 cells (22). *Malat1* expression was significantly correlated with a higher number of genes in in vitro–differentiated Th2 cells (1946) than in Th1 cells from the *Pc*AS-infected mice. As in the case of Th1 cells, the majority of statically significant correlations between expression of *Malat1* and other genes were negative (1455 out of 1946, 74.8%). Similarly, to the observed results with Th1 cells, there was again a positive correlation between *Malat1* and *Neat1* (Supplemental Fig. 2C). Of note, *Neat1* was the only gene neighboring *Malat1* that showed a significant correlation with *Malat1* in both Th1 and Th2 cells (Supplemental Fig. 2D), suggesting that *Malat1* has limited in cis regulatory effects in Th cells. This was further supported by expression analyses in *WT* and *Malat1*^−/−^ cells for *Neat1* and *Scyl1*, the two chromosomally adjacent genes to *Malat1*. *Neat1* levels were lower in naive *Malat1*^−/−^ CD4^+^ T cells, and *Scyl1* levels were higher in in vitro–differentiated *Malat1*^−/−^ Th2 cells, but these effects were not consistent among the three cell types (Supplemental Fig. 2E). As in the case of single Th1 cells, gene ontology analysis of the negatively correlated genes revealed again significant enrichment in genes involved in RNA binding, ribosomal function, metabolism, and cell cycle (Fig. 2F). There were 152 genes that were significantly correlated with *Malat1* in both single Th1 and Th2 cell–formed functional clusters with roles in RNA processing, ribosomal function, metabolism, and protein degradation (Fig. 2G, Supplemental Fig. 2F). Overall, these results indicated that, at the single-cell level, *Malat1* suppression in Th cells correlated with induction of key gene networks upon CD4^+^ T cell activation.

### *Malat1* controls IL-10 expression in Th cells in vitro

**FIGURE 3.**
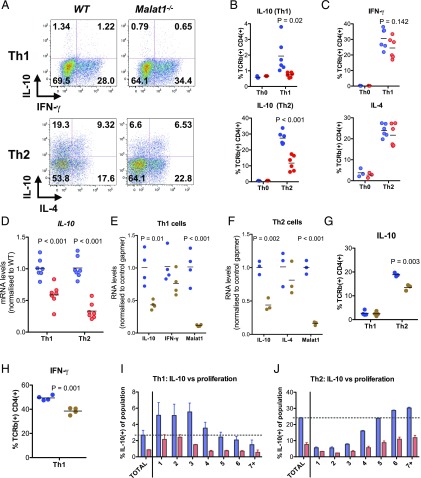
Loss of *Malat1* in in vitro–differentiated Th1 and Th2 cells results in suppression of IL-10 expression. (**A**) Representative FACS plots of IL-10 and IFN-γ or IL-10 and IL-4 expression in in vitro–differentiated Th1 and Th2 cells (day 6), respectively. (**B**) Percentage of IL-10^+^ live TCRβ^+^ CD4^+^ *WT* (blue) or *Malat1*^−/−^ (red) Th1 or Th2 cells. Levels determined by intracellular cytokine staining. Levels in Th0 cells shown for reference (*n* = 6 for Th1 and Th2, and *n* = 3 for Th0). (**C**) Percentage of IFN-γ^+^ or IL-4^+^ live TCRβ^+^ CD4^+^ *WT* (blue) or *Malat1*^−/−^ (red) Th1 or Th2 cells, respectively. Levels determined by intracellular cytokine staining. Levels in Th0 cells shown for reference (*n* = 6 for Th1 and Th2, and *n* = 3 for Th0). (**D**) *IL-10* mRNA levels in in vitro–differentiated *WT* (blue) or *Malat1*^−/−^ (red) Th1 and Th2 cells (day 6) determined by qRTPCR. Levels normalized to U6 and average levels in *WT* cells. (**E**) *IL-10*, *IFN-γ*, and *Malat1* RNA levels in in vitro–differentiated Th1 cells (day 6) transfected with control (blue) or *Malat1*-targeting (brown) gapmer (100 nM). Levels normalized to U6 and average levels in cells transfected with control gapmer. (**F**) *IL-10*, *IL-4*, and *Malat1* RNA levels in in vitro–differentiated Th2 cells (day 6) transfected with control (blue) or *Malat1*-targeting (brown) gapmer (100 nM). Levels normalized to U6 and average levels in cells transfected with control gapmer. (**G**) Percentage of IL-10^+^ live TCRβ^+^ CD4^+^ Th1 or Th2 cells transfected with control (blue) or *Malat1*-targeting (brown) gapmer (100 nM). Levels determined by intracellular cytokine staining on day 6. (**H**) Percentage of IFN-γ^+^ live TCRβ^+^ CD4^+^ Th1 cells transfected with control (blue) or *Malat1*-targeting (brown) gapmer (100 nM) (*n* = 4). (**I**) Percentage of IL-10^+^ CD4^+^ *WT* (blue) or *Malat1*^−/−^ (red) Th1 cells (day 6) per cell division as determined by intracellular cytokine and CFSE staining (*n* = 4). (**J**) Percentage of IL-10^+^ CD4^+^ *WT* (blue) or *Malat1*^−/−^ (red) Th2 cells (day 6) per cell division as determined by intracellular cytokine and CFSE staining (*n* = 4).

Having found that *Malat1* suppression is a hallmark of Th activation, we tested the effect of *Malat1* deletion on Th activation. We found that following in vitro differentiation of naive CD4^+^ T cells to Th1, *Malat1*^−/−^ cells displayed a reduction in levels of IFN-γ that did not reach statistical significance but significantly reduced expression of the immunoregulatory cytokine IL-10. Upon Th2 differentiation, there was also a significant reduction in IL-10 levels, with IL-4 being unaffected (Fig. 3A–C). The effect on IL-10 was more prominent in Th2 cells, which express higher levels of IL-10 than Th1 cells in vitro (Fig. 3A, 3B). We also observed a reduction in IL-10 mRNA levels (Fig. 3D). We repeated these experiments under weakly polarizing conditions and found a statistically significant reduction upon *Malat1* loss on IFN-γ expression in suboptimally activated Th1 cells but no effects on IL-10 or IL-4 and Il-10 under weakly polarizing Th2 conditions (Supplemental Fig. 3A). *Malat1* loss also suppressed IL-10 and IL-17 expression under Th17-differentiation conditions (Supplemental Fig. 3B). In addition to genetic knockout of *Malat1*, targeting the RNA product of *Malat1* with antisense oligonucleotide gapmers also suppressed IL-10 at the mRNA level in both Th1 and Th2 cells (Fig. 3E, 3F). The effect was also observed at the protein level in Th2 cells (Fig. 3G). In Th1 cells, gapmer-mediated inhibition of *Malat1* resulted in a significant decrease in IFN-γ expression with no effects on IL-10 (Fig. 3H). Notably, we found that the suppression of IL-10 expression occurred in *Malat1*^−/−^ CD4^+^ T cells irrespectively of how many times they have divided (Fig. 3I, 3J), demonstrating that the effect of *Malat1* on IL-10 is decoupled from Th cell proliferation. Overall, these results demonstrated that *Malat1* deficiency results in altered effector Th cell differentiation and cytokine expression in vitro, with a common effect among Th1, Th2, and Th17 cells being suppression of IL-10.

### Loss of *Malat1* impairs Maf expression in Th cells

**FIGURE 4.**
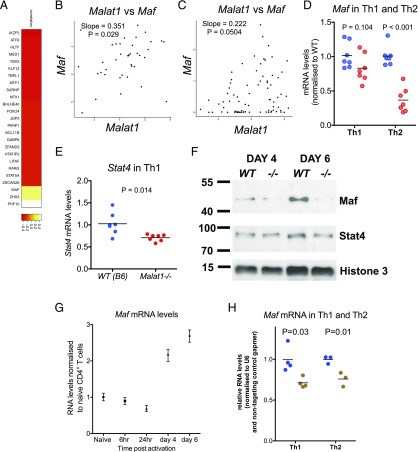
*Malat1* regulates Maf in Th cells. (**A**) Heatmap of correlation coefficients of significantly correlated transcription factors and coactivators (*p* < 0.05) and *Malat1* expression by single PbTII cells isolated from *Pc*AS-infected mice at day 7 p.i. (**B**) Normalized transcript count of *Malat1* versus *Maf* in single PbTII cells isolated from *Pc*AS-infected mice at day 7 p.i. (**C**) Normalized transcript count of *Malat1* versus *Maf* in single in vitro–differentiated Th2 cells. (**D**) *Maf* mRNA levels in in vitro–differentiated *WT* (blue) or *Malat1*^−/−^ (red) Th1 and Th2 cells (day 6) determined by qRTPCR. (**E**) *Stat4* mRNA levels in in vitro–differentiated *WT* (blue) or *Malat1*^−/−^ (red) Th1 cells (day 6). In (D) and (E), levels are determined by qRTPCR (*n* = 7) and normalized to U6 and average levels in *WT* cells. (**F**) Maf and Stat4 protein levels in *WT* (blue) or *Malat1*^−/−^ (red) Th1 cells at days 4 or 6 postactivation determined by Western blot. (**G**) *Maf* mRNA levels during in vitro Th1 differentiation, normalized to naive CD4^+^ T cells. Levels normalized to U6 and average levels in naive CD4^+^ T cells. (**H**) *Maf* mRNA levels in Th1 or Th2 cells transfected with control (blue) or *Malat1*-targeting (brown) gapmer (100 nM). Levels normalized to U6 and average levels in cells transfected with control gapmer.

To explore the mechanism employed by *Malat1* to regulate IL-10, we determined the effect of *Malat1* on transcription factors associated with Th cell activation and differentiation. Analysis of our single-cell RNA-seq data revealed 25 transcription factors that showed a significant correlation with *Malat1* expression in Th1 cells from *Pc*AS-infected mice (Fig. 4A). Notably, one of only three transcription factors that showed positive correlation with *Malat1* was *Maf* (also known as *c-Maf*; Fig. 4B), a protein with fundamental roles in CD4^+^ T cell biology and, notably, an essential transcriptional regulator of IL-10 expression in Th cells (23–28). We also observed a positive correlation between *Malat1* and *Maf* in single Th2 cells, which was borderline statistically nonsignificant (*p* = 0.0504; Fig. 4C). Despite a lack of a positive correlation between *Malat1* and IL-10 in our single-cell analyses, possibly because of the low number of IL-10–expressing cells in the analyzed datasets (Supplemental Fig. 3C), *Maf* was one of only five genes positively correlating with expression of IL-10 and *Malat1* in single *Plasmodium*-specific CD4^+^ T PbTII cells (Supplemental Fig. 3D). Investigating a list of previously published transcription factors correlating with IL-10 expression in CD4^+^ T cells (23) for overlap with genes correlated with *Malat1* in single CD4^+^ T PbTII cells also identified *Maf* as the only gene present in both sets.

Next, we tested the effect of lack of *Malat1* on levels of transcription factors with key roles in Th cell differentiation. *Malat1* did not affect levels of *Tbet* in Th1 cells or *Gata3* in Th2 cells (Supplemental Fig. 3E, 3F). However, *Maf* levels were reduced in both Th1 and Th2 cells, the effect reaching statistical significance in Th2 cells (Fig. 4D), which express higher levels of *Maf* than Th1 cells in vitro (Supplemental Fig. 3G). In *Malat1*^−/−^ Th1 cells, we also observed a significant downregulation of *Stat4* (Fig. 4E), with no changes observed in *Stat6* in Th2 cells (Supplemental Fig. 3H). Of note, Stat4 is also known to promote IL-10 transcription in Th1 cells (29) and was also downregulated in *Malat1^−/−^* Th1 cells. At the protein level, we observed only a modest suppression of Stat4 expression in *Malat1^−/−^* Th1 cells at day 6 (Fig. 4F), whereas suppression of Maf was more pronounced than that observed at the mRNA level (Fig. 4D). Of note, the kinetics of *Maf* mRNA levels in Th1 cells demonstrated an early reduction followed by an increase (Fig. 4G), suggesting that *Malat1* might be playing a role in Maf regulation at both the early and later stages of Th1 differentiation. Knockdown of *Malat1* with gapmers suppressed *Maf* levels in both Th1 and Th2 cells, demonstrating that the effect is mediated by the *Malat1* RNA (Fig. 4H). Furthermore, levels of Bhlhe40, a transcription factor involved IL-10 regulation (30) and anticorrelating with *Malat1* (Fig. 4A), were not affected in *Malat1^−/−^* Th1 or Th2 cells (Supplemental Fig. 3I). Taken together, the above demonstrate that *Malat1* loss suppresses expression of Maf, a central transcriptional regulator of IL-10 in Th cells.

### *Malat1*^−/−^ mice infected with *L. donovani* demonstrate reduced IL-10 expression in CD4^+^ T cells and lower parasite burden

**FIGURE 5.**
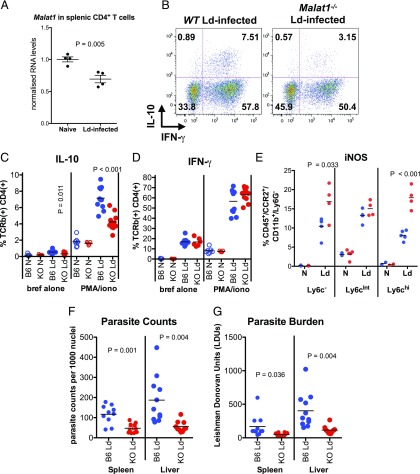
Loss of *Malat1* results in suppression of IL-10 expression in Th1 cells in vivo and enhanced immunity to *L. donovani*. (**A**) *Malat1* levels determined by qRTPCR in naive (N) CD4^+^ T cells and CD4^+^ T cells isolated from spleens of *L. donovani*–infected mice (day 28) p.i. Levels normalized to U6 and average levels in N CD4^+^ T cells. (**B**) Representative FACS plots of IL-10 and IFN-γ expression in splenic CD4^+^ T cells from *L. donovani*–infected *WT* and *Malat1*^−/−^ mice (day 42 p.i.) following restimulation with PMA/ionomycin. (**C**) Percentage of IL-10^+^ live TCRβ^+^ CD4^+^ cells from *L. donovani*–infected *WT* (blue) or *Malat1*^−/−^ (red) mice (day 42 p.i.), determined by intracellular cytokine staining. Levels from noninfected N mice are also shown for reference. Levels are shown in cells directly postisolation (brefeldin A [bref] alone) or following restimulation with PMA and ionomycin (PMA/iono). (**D**) Percentage of IFN-γ^+^ live TCRβ^+^ CD4^+^ cells from *L. donovani* infected *WT* (blue) or *Malat1*^−/−^ (red) mice (day 42 p.i.), determined by intracellular cytokine staining. Levels from noninfected N mice are also shown for reference. Levels are shown in cells directly postisolation (bref alone) or following restimulation with PMA/iono. (**E**) Percentage of inducible NO synthase-positive (iNOS^+^) myeloid live cells from spleens of noninfected (N) or *L. donovani*–infected *WT* (blue) or *Malat1*^−/−^ (red) mice (day 42 p.i.; *n* = 5). (**F**) Spleen and liver parasite counts per 1000 nuclei from *L. donovani*–infected *WT* (blue) or *Malat1*^−/−^ (red) mice (day 42 p.i.). (**G**) Spleen and liver Leishman–Donovan units from *L. donovani*–infected *WT* (blue) or *Malat1*^−/−^ (red) mice (day 42 p.i.). For (C), (D), (F), and (G), *n* = 11, and for (E), *n* = 5.

Next, to explore the functional role of *Malat1*/Maf/IL-10 pathway in vivo, we studied the role of *Malat1* in infection models in which IL-10 deficiency either promotes pathogen clearance or enhances immunopathology. First, we used *L. donovani* infection of mice as a model of pathogen-induced chronic inflammation (31). We chose to study a model of visceral leishmaniasis because Th1 cell–derived IL-10 has been previously shown to be a critical for protection and pathogen clearance but also a critical determinant of disease outcomes in humans (16, 32–36). *Malat1* expression was reduced in CD4^+^ T cells isolated from spleens of infected mice compared with naive CD4^+^ T cells (Fig. 5A). Critically, we found that at the chronic infection stages (day 42 p.i.), IL-10 expression was reduced in splenic CD4^+^ T cells from infected *Malat1*^−/−^ mice without any changes in IFN-γ expression (Fig. 5B–D), mirroring our findings from in vitro–activated CD4^+^ T cells (Fig. 4). There were no statistically significant changes in the number of splenic CD4^+^ T cells or frequency of naive (CD62L^+^/CD44^−^) and effector cells (CD62L^−^/CD44^+^) CD4^+^ T cells between *WT* and *Malat1*^−/−^ mice (Supplemental Fig. 4A, 4B). The reduction in IL-10^+^ Th1 cells was accompanied with increased inducible NO synthase expression from splenic myeloid cells, particularly CD11b^+^/CCR2^+^/Ly-6C^hi^ inflammatory monocytes in *Malat1*^−/−^ mice (Fig. 5E). No changes in MHCII or IL-10 were found in any of the myeloid populations (Supplemental Fig. 4C, 4D). Critically, the observed reduction in IL-10 levels was accompanied by significantly reduced parasite loads in *Malat1*^−/−^ mice (Fig. 5F, 5G) without any significant effects on spleen or liver size (Supplemental Fig. 4E). These results demonstrated that *Malat1* regulates IL-10 in Th cells in vivo and that *Malat1* deficiency can lead to enhanced protection during chronic *L. donovani* chronic infection.

### *Pc*AS-infected *Malat1*^−/−^ mice demonstrate reduced Th1 IL-10 expression and more severe disease

**FIGURE 6.**
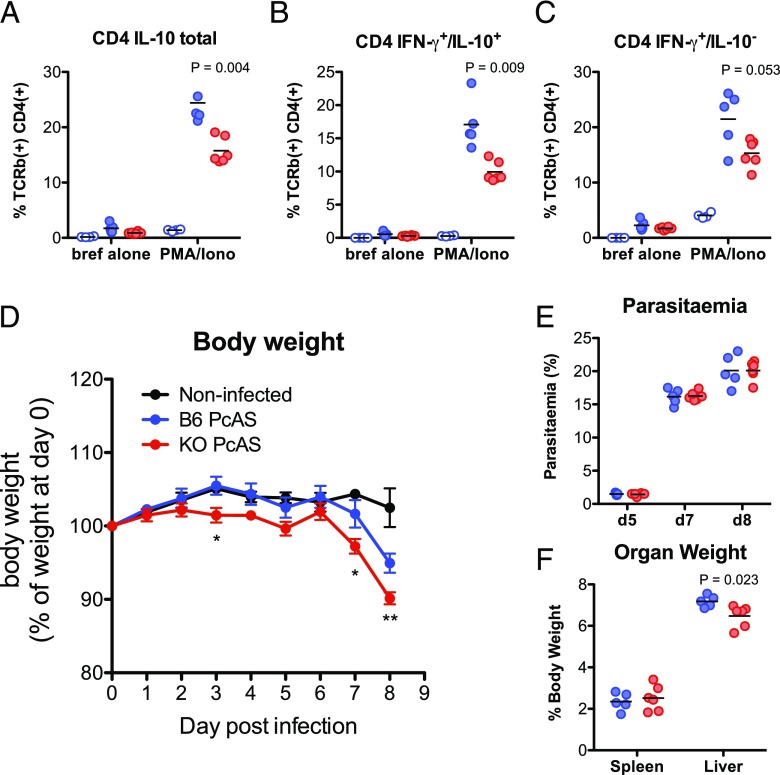
Loss of *Malat1* results in suppression of IL-10 expression in Th1 cells and more severe disease in *Pc*AS-infected mice. (**A**) Percentage of IL-10^+^ live TCRβ^+^ CD4^+^ cells from *Pc*AS-infected *WT* (blue) or *Malat1*^−/−^ (red) mice (day 8 p.i.), determined by intracellular cytokine staining. Levels from noninfected naive (N) mice are also shown for reference. Levels are shown in cells directly postisolation (brefeldin A [bref] alone) or following restimulation with PMA and ionomycin (PMA/iono). (**B**) As in (A) but for percentage of IFN-γ^+^/IL-10^+^ live TCRβ^+^ CD4^+^ cells from *Pc*AS-infected *WT* (blue) or *Malat1*^−/−^ (red) mice (day 8 p.i.). (**C**) As in (A) but for percentage of IFN-γ^−^/IL-10^+^ live TCRβ^+^ CD4^+^ cells from *Pc*AS-infected *WT* (blue) or *Malat1*^−/−^ (red) mice (day 8 p.i.). (**D**) Body weight of *Pc*AS-infected *WT* (B6, blue) or *Malat1*^−/−^ (red) mice on indicated days p.i. Weight expressed as percentage of starting weight for each mouse. Black line shows weights of noninfected mice. *p < 0.05, **p < 0.01, between infected *WT* and *Malat1*^−/−^ mice. (**E**) Parasitemia (percentage of infected cells) in *Pc*AS-infected *WT* (B6, blue) or *Malat1*^−/−^ (red) mice on indicated days p.i. (**F**) Spleen and liver weights as percentage of body weight in *Pc*AS-infected *WT* (B6, blue) or *Malat1*^−/−^ (red) mice at day 8 p.i. For (A)–(F), *n* = 4–6.

To further test the functional relevance of *Malat1*-mediated IL-10 regulation in vivo, we infected *Malat1*^−/−^ and *WT* mice with *Pc*AS. IL-10 plays a prominent role in the outcome of malaria disease in humans (37, 38), and in the *Pc*AS experimental model of malaria, IL-10 deficiency promotes severe disease manifested as more pronounced weight loss and mortality (39). As in the case of *L. donovani* infection, CD4^+^ T cells from spleens of *Pc*AS-infected *Malat1*^−/−^ mice demonstrated lower IL-10 expression (Fig. 6A, 6B). A borderline nonsignificant trend toward reduction in IFN-γ^+^ CD4^+^ T cells was also observed (Fig. 6C). Crucially, more pronounced weight loss was observed in *Malat1*^−/−^ mice compared with *WT* controls within the first week of *Pc*AS infection (Fig. 6D). Of note, the experiment had to be terminated because of the increased rate of weight loss in *Malat1*^−/−^ mice (expected to exceed 20% of starting body weight by day 9) and lack of touch escape and pinna reflexes in *Malat1*^−/−^ at day 8 (median score of 0 out of 2 for both). We did not observe any effects on parasitemia (Fig. 6E). Spleen enlargement was similar between *WT* and *Malat1*^−/−^ mice, but a modest reduction liver size in *Pc*AS-infected *Malat1*^−/−^ mice was observed (Fig. 6F). Of note, *Malat1* did not affect IL-10 and IFN-γ levels in CD8^+^ T cells in infected mice (Supplemental Fig. 4F, 4G). The above findings further demonstrated the role of *Malat1* in regulation of IL-10 in Th cells in vivo and supported that the extent of *Malat1* downregulation and its expression kinetics during *Plasmodium* infections can be a significant determinant of infectious disease outcome.

## Discussion

Despite its high abundance and conservation, the physiological function of *Malat1* at the whole organism level has remained elusive. We demonstrate that regulation of adaptive immunity is one of the essential functions of this unique noncoding RNA. We show that suppression of *Malat1*, one of the most highly expressed transcripts in naive CD4^+^ T cells, is a hallmark of Th1 and Th2 activation, but its complete deletion results in altered Th cell phenotype and enhanced Th cell responses in vivo, which can lead to protection from infection but also severe immunopathology. It is possible that suppression of *Malat1* can be due to dilution occurring during the initial transcriptional burst at the early stages of naive CD4^+^ T cell activation. This would mean that there are transcriptional mechanisms excluding *Malat1*, a very highly expressed and transcribed transcript, from this general burst. We note that these mechanisms would be specific to *Malat1*, as we show that the expression of other highly expressed lncRNAs is not altered or diluted upon naive CD4^+^ T cell activation (Fig. 1C). Furthermore, *Malat1* suppression is sustained up to 6 d postactivation, suggesting the existence of active transcriptional suppression and/or posttranscriptional destabilization mechanisms regulating its expression in Th cells. The observed inverse correlation between *Malat1* expression and transcriptional signatures associated with cellular activation in single Th cells might signify that high *Malat1* expression is necessary for maintenance of the naive CD4^+^ T cell state or that suppressed *Malat1* expression is required for appropriate Th cell differentiation. The seeming contradiction between our findings and those reported by Yao and colleagues (14) during acute LCMV infection can be explained by the predominant focus of that study on CD8^+^ T cell responses and the fact that IL-10 determines susceptibility to infection only in chronic LCMV infection models (40). In our study, the effect of *Malat1* on IL-10 expression is observed both in in vitro differentiated Th cells and in vivo in two distinct infection models progressing at significantly different timescales (days for *Pc*AS versus weeks for *L. donovani*), demonstrating a CD4^+^ T cell–intrinsic regulatory role for *Malat1*. We note that because of the use of a full *Malat1*^−/−^ mouse, we cannot exclude other CD4^+^ T cell–independent mechanism contributing to *L. donovani* clearance or *Pc*AS-induced immunopathology. However, we propose that despite its widespread expression across tissues, *Malat1* has striking CD4^+^ T cell–specific functions, one of which involves promoting Maf and IL-10 expression.

It is thought that *Malat1* controls gene expression through interacting with multiple RBPs (9, 21, 41, 42). Our experiments with *Malat1* inhibitors confirmed that the *Malat1* RNA is responsible for altered Th cell differentiation. We note that we observed slight differences between the effect of *Malat1* knockout and that of knockdown in Th1 cells (e.g., with regards to IFN-γ expression). These observations can be the result of the fact that *Malat1* is deleted in naive CD4^+^ T *Malat1^−/−^* cells before they are activated, whereas in cells treated with gapmers, depletion occurs concurrently with activation and endogenous downregulation of *Malat1*. In any case, we show that inhibiting or deleting *Malat1* can lead to reduction of IL-10 and Maf expression in vitro and in vivo. We propose that the specificity of *Malat1* functions in CD4^+^ T cells is mediated through a network of interactions between *Malat1* and RBPs, which, in turn, have cell-type specific RNA targets and functions (43). Indeed, we anticipate that *Malat1* functions in Th cell differentiation are likely to extend beyond regulation of Maf and IL-10. The work presented in this study reveals one mechanism employed by *Malat1* to shape immunity to infection and indicates that this lncRNA, like IL-10, plays a critical role in controlling the fragile equilibrium between effective pathogen clearance and enhanced immunopathology. Our work suggests that exploring how *Malat1*-binding RBPs regulate Maf and IL-10 expression through *Malat1*-dependent or -independent mechanisms can provide key insight into the posttranscriptional regulation of this critical immunoregulatory axis.

Implicating *Malat1* in the regulation of IL-10 can have far-reaching consequences. Although all activated (effector and regulatory) CD4^+^ T cells express IL-10 at some point of their differentiation trajectory (26), the magnitude and kinetics of expression differ drastically between subsets. Yet, despite significant progress on the transcriptional cues that initiate and maintain IL-10 expression in CD4^+^ T cells, much less is known about the molecular controllers that ensure accurate timing and magnitude of IL-10 expression. Our work supports that *Malat1* plays a key role in the complex process that ensures optimal IL-10 levels. Notably, the high abundance of *Malat1* in naive CD4^+^ T cells is evolutionary-compatible with this role. Although *Malat1* expression is reduced early during CD4^+^ T cell activation, its high absolute abundance means that there is still a significant *Malat1* pool within effector Th cells to allow for appropriate IL-10 expression. This provides a flexible molecular mechanism of regulating immune responses mediated by *Malat1*, a major component of the CD4^+^ T cell noncoding transcriptome. Although predominantly focused on Th1 and Th2 cells, our work suggests that *Malat1* might be a significant functional determinant of other Maf- and IL-10–expressing immune cell types, such as regulatory T cells (44), follicular Th cells (45, 46), or innate lymphoid cells (47).

Overall, our findings reveal *Malat1* as a negative regulator of immunity to infection. We uncover the functional significance of *Malat1* in the context of two major parasitic infectious diseases, malaria and visceral leishmaniasis, providing new insight into molecular determinants of disease susceptibility. We speculate that through its fundamental role in Th cell differentiation and function, *Malat1* is likely to govern immune responses and disease outcomes in a broad range of infectious, autoimmune, or inflammatory pathological conditions, reflecting the centrality of the noncoding transcriptome in the immune system.

## Supplementary Material

Data Supplement
