# Supplementary material for: Malat1 Suppresses Immunity to Infection through Promoting Expression of Maf and IL-10 in Th Cells
Source: J Immunol. 2020 Apr 22;204(11):2949–60. doi: 10.4049/jimmunol.1900940 (PMC7231852; doi:10.4049/jimmunol.1900940)
Supplement: Data Supplement [file JI_1900940.zip › JI_1900940_Supplemental_Figures_1.pdf]

Supplemental Fig. 1

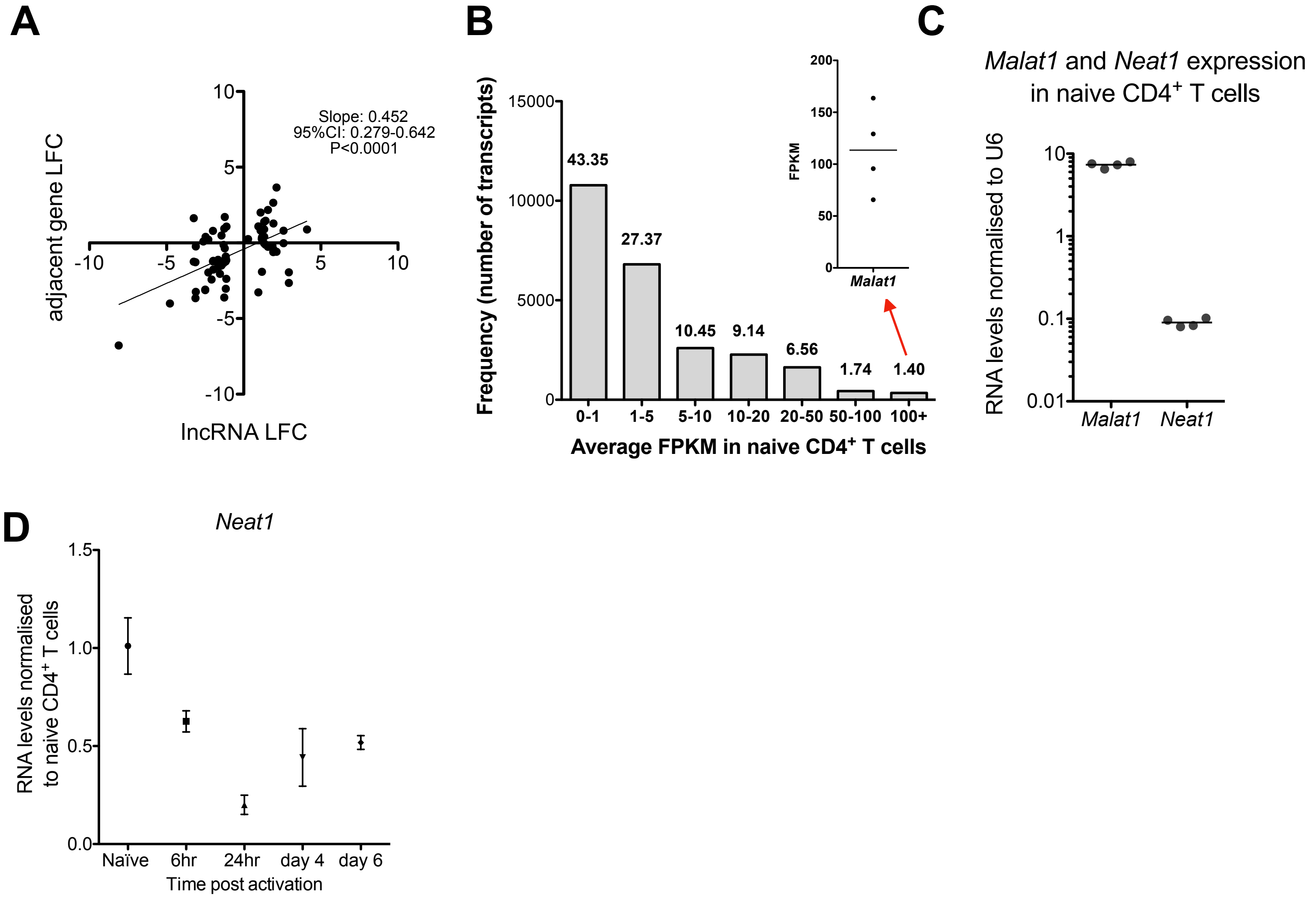

Supplemental Figure 1: Identification of differentially regulated lncRNAs upon activation of naïve CD4+ T cells reveals *Malat1* suppression as a hallmark of Th cell activation.

A. LFC of lncRNAs vs LFC of adjacent genes. For each lncRNA LFC of its two adjacent genes is shown. P value indicates significance of slope being statistically significantly different from 0 (linear regression).

B. Frequency of transcripts based on their expression (FPKM) in naïve CD4+ T cells (all transcripts). Expression determined by bulk RNA-seq. Numbers on top of the bars show percentage of transcripts with this level of expression. Inset shows *Malat1* levels.

C. *Malat1* and *Neat1* levels in naïve CD4+ T cells normalised to U6 RNA (n=4).

D. *Neat1* expression during *in vitro* Th1 differentiation, normalised to naïve CD4+ T cells.

Supplemental Fig. 2

A

LncRNA	*Malat1*	*Lncpint*
**Significantly correlated genes**	687	452
**Negatively correlated**	609	22
**Positively correlated**	78	430

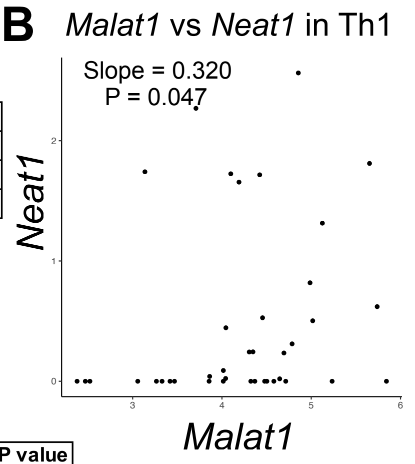

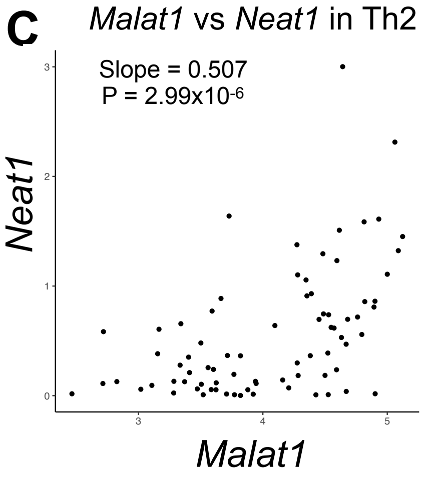

D

Gene Symbol	Position	Th1 P value	Th2 P value
Rnaseh2c	chr19:5601873-5603439	0.035	0.139
Kat5	chr19:5603017-5610094	0.335	0.652
Rela	chr19:5637483-5648130	0.976	0.420
Sipa1	chr19:5651185-5663707	0.050	0.280
Pcnx3	chr19:5664635-5688908	nd	nd
Map3k11	chr19:5689131-5702862	0.200	0.415
Kcnk7	chr19:5704367-5707101	0.122	0.588
Ehbp1l1	chr19:5707376-5726317	0.014	0.242
Fam89b	chr19:5728087-5729651	0.541	nd
Ltbp3	chr19:5740904-5758532	nd	0.655
Scyl1	chr19:5758427-5771401	0.197	0.886
**Malat1**	chr19:5795690-5802672	**0**	**0**
Neat1	chr19:5824708-5845478	0.047	<0.001
Frmd8	chr19:5849702-5875274	0.667	0.024
Slc25a45	chr19:5877817-5885766	0.752	0.215
Tigd3	chr19:5891139-5894107	0.133	0.539
Dpf2	chr19:5896516-5912871	0.095	0.762
Cdc42ep2	chr19:5917556-5924816	nd	0.172
Pola2	chr19:5940543-5964206	0.441	0.060
Slc22a20	chr19:5970234-5986143	nd	nd
Capn1	chr19:5988546-6015247	0.558	0.958

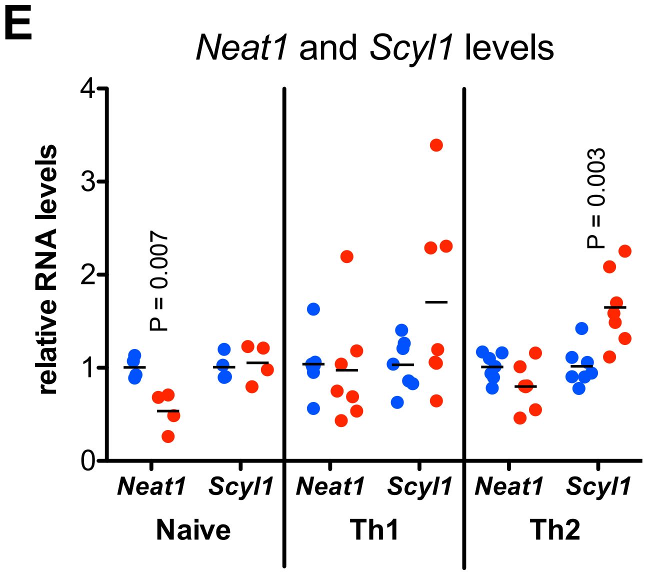

F

Cluster 1: Protein degradation	Cluster 2: Transcription	Cluster 3: RNA-binding and processing	Cluster 4: Cell structure and localisation	Cluster 5: Translation and ribosomes	Cluster 6: Metabolism
Gm4950	Casc5	Gm5616	Actb	Eif4a1	Atp5g3
Nelfcd	Cbx3	Gm8186	Actg1	Eif4a3	Cox5a
Psma6	Dpy30	Hnrnpa1	Arpc5	Eif4g1	Ndufa12
Psma7	H2afz	Hnrnpa2b1	Dctn5	Eif5a	Ndufaf2
Psmb3	Hdac1	Hnrnpu	Fkbp5	Ict1	Ndufaf5
Psmb6	Prmt5	Noc4l	Hsp90ab1	Mrpl12	
Psmc1		Prpf31	Rab1a	Mrpl2	
Psmc3		Prpf38a	Stip1	Mrpl20	
Psmd2		Prpf40a	Surf4	Mrpl35	
Psmd8		Puf60		Mrpl37	
		Sf3b2		Mrps34	
		Snrnp40		Rpl5	
		Snrpb		Rps27l	
		Snrpg		Slc35b1	
		Srsf7			
		Utp3			

Supplemental Figure 2: Transcriptional units and transcription factors correlating with *Malat1* expression at single cell level in Th cells *in vivo*.

A. Total number of genes showing a significant correlation with *Malat1* or *Lncpint* in single PbTII cells isolated from *Pc*AS-infected mice 7 days p.i. Number of genes showing positive or negative correlation also shown.

B. Normalised transcript count of *Malat1* versus *Neat1* in single PbTII cells isolated from *Pc*AS-infected mice 7 days p.i.

C. Normalised transcript count of *Malat1* versus *Neat1* in single *in vitro* differentiated Th2 cells.

D. Correlation coefficients and P values for *Malat1* and genes in its genomic neighbourhood (400kb region around the *Malat1* locus) in single PbTII cells isolated from *Pc*AS-infected mice 7 days p.i.

E. RNA levels of *Neat1* and *Scyl1* in *WT* (blue) or *Malat1*$^{-/-}$ (red) naïve, Th1, and Th2 cells. Levels are normalised to U6 and average levels in *WT* cells for each condition. Levels determined by qRTPCR; n=4 for naïve and n=7 for Th1 and Th2 cells. Significant P values shown.

F. Genes names for genes shown in **Fig. 2G** and their clusters.

Supplemental Fig. 3

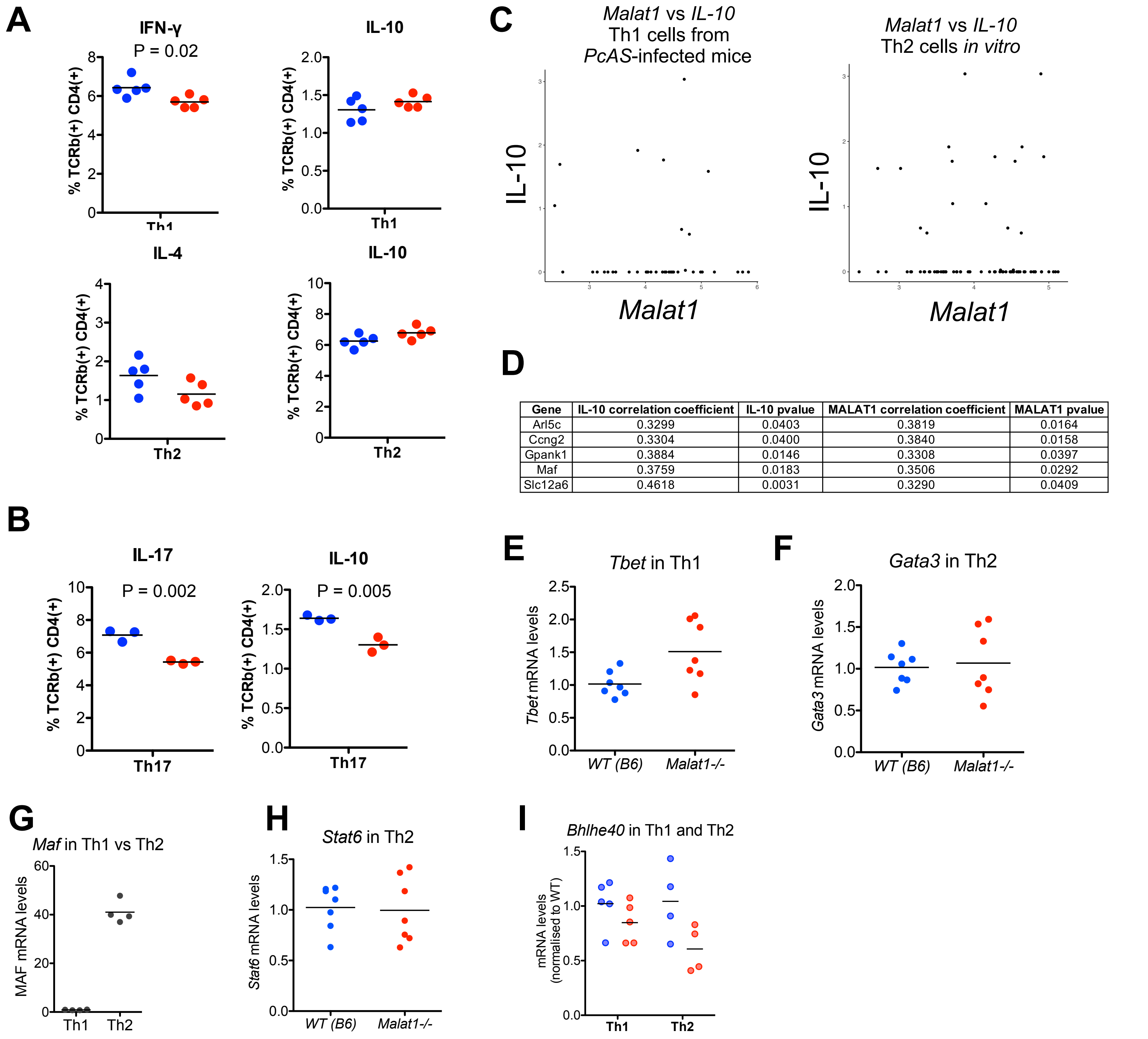

Gene	IL-10 correlation coefficient	IL-10 pvalue	MALAT1 correlation coefficient	MALAT1 pvalue
Arl5c	0.3299	0.0403	0.3819	0.0164
Ccng2	0.3304	0.0400	0.3840	0.0158
Gpank1	0.3884	0.0146	0.3308	0.0397
Maf	0.3759	0.0183	0.3506	0.0292
Slc12a6	0.4618	0.0031	0.3290	0.0409

Supplemental Figure 3: Effect of Malat1 deletion on key Th cell transcription factors

A. Percentage of IL-10⁺, IFN-γ⁺ or IL-4⁺ live TCRβ⁺ CD4⁺ WT (blue) or Malat1$^{-/-}$ (red) under conditions inducing suboptimal Th1 or Th2 differentiation (see Materials and Methods). Levels determined by intracellular cytokine staining. N=5.

B. Percentage of IL-10⁺ or IL-17⁺ live TCRβ⁺ CD4⁺ WT (blue) or Malat1$^{-/-}$ (red) in vitro differentiated Th17 cells. Levels determined by intracellular cytokine staining. N=3.

C. Normalised transcript count of Malat1 versus IL10 in single PbTII Th1 cells (left graph) and normalised transcript count of Malat1 versus IL10 in single Th2 cells (right graph).

D. Correlation coefficients and P values for genes significantly correlating with both Malat1 and IL-10 in single PbTII cells from PcAS-infected mice 7 days p.i.

E. Tbet mRNA levels in WT (blue) or Malat1$^{-/-}$ (red) Th1 cells.

F. Gatat3 mRNA levels in WT (blue) or Malat1$^{-/-}$ (red) Th2 cells.

G. Maf mRNA levels in WT Th1 and Th2 cells (day 6) determined by qRTPCR. Levels normalised to U6 and average levels in Th1 cells.

H. Stat6 mRNA levels in in vitro differentiated WT (blue) or Malat1$^{-/-}$ (red) Th1 cells (day 6).

I. Bhlhe40 mRNA levels in WT (blue) or Malat1$^{-/-}$ (red) Th1 or Th2 cells.

For **C**, **D**, and **F**, levels are determined by qRTPCR (n=7) and normalised to U6 and average levels in WT cells.

Supplemental Fig. 4

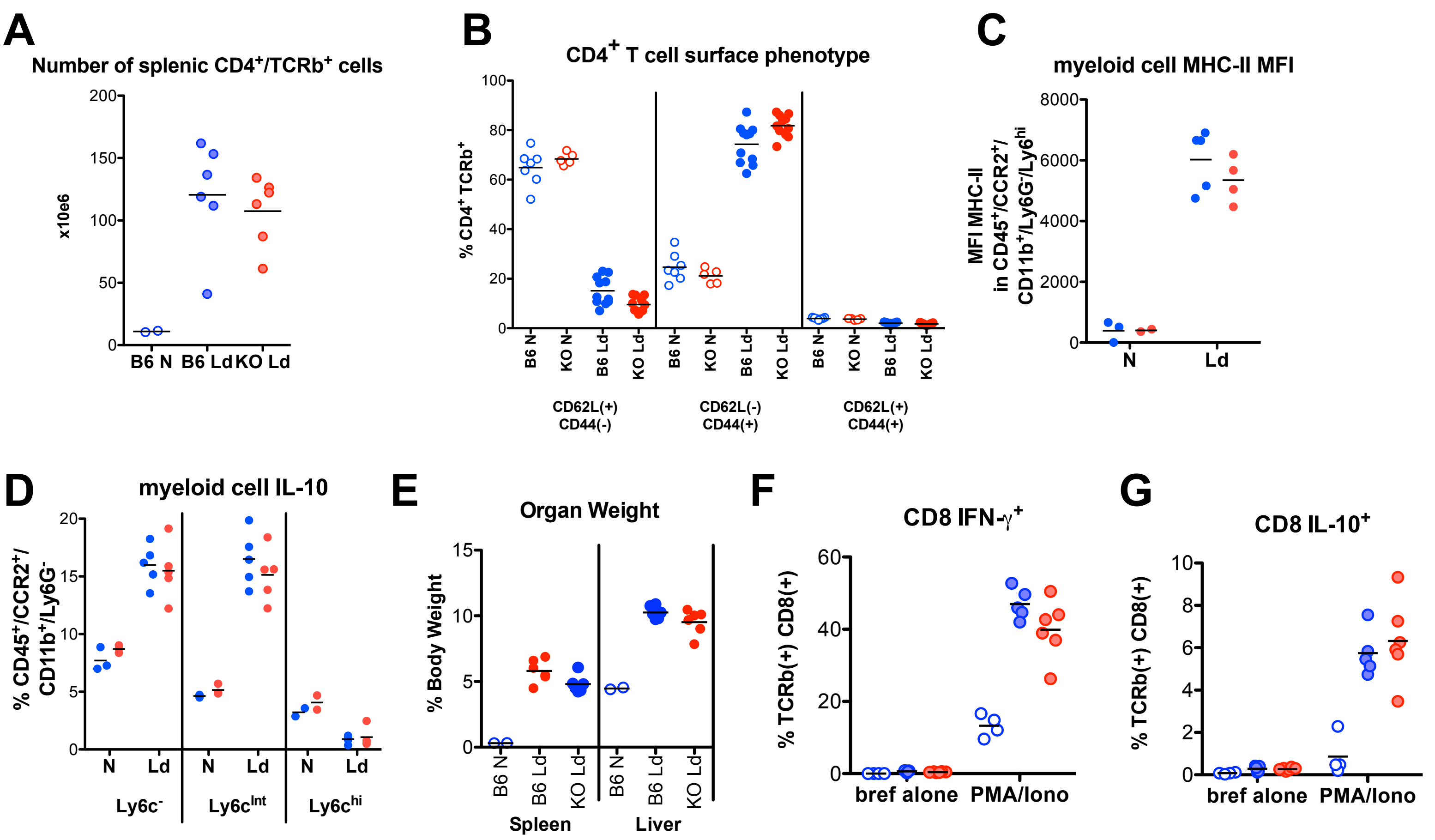

Supplemental Figure 4: Loss of *Malat1* results in suppression of IL-10 expression in Th1 cells *in vivo* and enhanced immunity to *Leishmania donovani* but more severe malaria

A. Number of splenic CD4+/ TCRβ + cells in spleens of non-infected *WT* mice (B6; empty blue circles, n=2) and *L. donovani* infected *WT* (blue) or *Malat1*-/- (red) mice (day 42 p.i; n=6).

B. Surface phenotype as determined by flow cytometry and CD62L and CD44 surface staining in non-infected *WT* (N) and *Malat1*-/- (N) mice (empty blue and red circles, respectively) and *L. donovani* infected *WT* (Ld) or *Malat1*-/- (Ld) mice (full blue and red circles, respectively; day 42 p.i, n=8-11).

C. Mean fluorescent intensity (MFI) of MHC-II expression on inflammatory monocytes from non-infected (N) and *L. donovani* infected (Ld) *WT* (blue) or *Malat1*-/- (red) mice.

D. Percentage of IL-10+ myeloid live cells from spleens of non-infected (N) or *L. donovani* infected (Ld) *WT* (blue) or *Malat1*-/- (red) mice (day 42 p.i, n=5).

E. Spleen and liver weight (as percentage of body weight) from *L. donovani* infected *WT* (blue) or *Malat1*-/- (red) mice (day 42 p.i). Naïve (N) non-infected mice are also shown for reference.

F. Percentage of IFN-γ+ TCRβ+ CD8+ cells from *Pc*AS infected *WT* (blue) or *Malat1*-/- (red) mice (day 8 p.i., n=4 – 6), determined by intracellular cytokine staining. Levels from non-infected naïve (N) mice are also shown for reference. Levels are shown in cells directly post-isolation (bref alone) or following restimulation with PMA and ionomycin (PMA/iono).

G. As in **F** but for percentage of IL-10+ TCRβ+ CD8+ cells from *Pc*AS infected *WT* (blue) or *Malat1*-/- (red) mice (day 8 p.i., n=4 – 6).
